# Inactivation of HIV-1 in Polarized Infant Tonsil Epithelial Cells by Human Beta-Defensins 2 and 3 Tagged with the Protein Transduction Domain of HIV-1 Tat

**DOI:** 10.3390/v13102043

**Published:** 2021-10-11

**Authors:** Rossana Herrera, Kristina Rosbe, Sharof M. Tugizov

**Affiliations:** 1 Department of Medicine, University of California–San Francisco, 513 Parnassus Ave., San Francisco, CA 94143, USA; bkrossana@gmail.com; 2 Department of Otolaryngology, University of California–San Francisco, San Francisco, CA 94115, USA; Kristina.Rosbe@ucsf.edu

**Keywords:** human immunodeficiency virus, human beta-defensins 2 and 3, tonsil epithelium, HIV-1 Tat, protein transduction domain, cell-penetrating peptides

## Abstract

Mother-to-child transmission (MTCT) of HIV-1 may occur during pregnancy, labor, and breastfeeding; however, the molecular mechanism of MTCT of virus remains poorly understood. Infant tonsil mucosal epithelium may sequester HIV-1, serving as a transient reservoir, and may play a critical role in MTCT. Innate immune proteins human beta-defensins 2 (hBD-2) and -3 may inactivate intravesicular virions. To establish delivery of hBD-2 and -3 into vesicles containing HIV-1, we tagged hBDs with the protein transduction domain (PTD) of HIV-1 Tat, which facilitates an efficient translocation of proteins across cell membranes. Our new findings showed that hBD-2 and -3 proteins tagged with PTD efficiently penetrated polarized tonsil epithelial cells by endocytosis and direct penetration. PTD-initiated internalization of hBD-2 and -3 proteins into epithelial cells led to their subsequent penetration of multivesicular bodies (MVB) and vacuoles containing HIV-1. Furthermore, PTD played a role in the fusion of vesicles containing HIV-1 with lysosomes, where virus was inactivated. PTD-initiated internalization of hBD-2 and -3 proteins into ex vivo tonsil tissue explants reduced the spread of virus from epithelial cells to CD4+ T lymphocytes, CD68+ macrophages, and CD1c+ dendritic cells, suggesting that this approach may serve as an antiviral strategy for inactivating intraepithelial HIV-1 and reducing viral MTCT.

## 1. Introduction

Mother-to-child transmission (MTCT) is an important pathway for the spread of human immunodeficiency virus (HIV) from mother to fetus, neonate, and infant. The precise molecular mechanisms of HIV MTCT remain unclear. HIV-1 transmigrates through fetal/infant oropharyngeal and tonsil mucosal epithelia, indicating a critical role of oral mucosal epithelium in HIV MTCT [1,2]. The majority (>90%) of virions internalized into vesicles of infant tonsil epithelium, including multivesicular bodies (MVB) and vacuoles, are sequestered in the endosomes for up to 9 days [3]. In contrast, such prolonged intracellular sequestration of HIV is not observed in adult tonsil epithelial cells, where intracellular virus is rapidly inactivated by high-level expression of the anti-HIV innate immune proteins human beta-defensin 2 (hBD-2) and hBD-3 [1,4]. However, the lack of, or low-level expression of hBD-2 and -3 in fetal and infant oral epithelial cells leads to the transmission of infectious HIV via fetal/infant oropharyngeal and tonsil epithelia [1,4].

HIV-1 sequestration has also been observed in vaginal, cervical, prostate, and kidney epithelial cells [5,6,7,8]. HIV-1 was detected in MVB of kidney epithelial cells by using transmission electron microscopy [6].

HIV-1 internalization into epithelial cells is initiated by multiple entry pathways, including clathrin-, caveolin/lipid raft-associated endocytosis, and macropinocytosis [9]. Attachment of HIV-1 to galactosylceramide and heparan sulfate proteoglycans of infant tonsil epithelial cells plays a critical role in HIV-1 endocytosis and macropinocytosis [9]. HIV-1 also binds to T-cell immunoglobulin and mucin domain 1 protein of polarized infant tonsil epithelial cells, which initiate viral macropinocytosis [9]. HIV endocytosis and macropinocytosis lead to sequestration of virions in late endosomes, including MVB and vacuoles [3,9].

MVB are late endosomes with intraluminal vesicles, which are formed by fission of early endosomal compartments from their cytoplasmic face using endosomal sorting complexes required for transport [10,11]. Maturation of early endosomes containing HIV into MVB may lead to sequestration of virus in the MVB. Homophilic fusion of vesicles containing HIV [12,13,14,15,16,17,18] may also lead to formation of large vacuoles sequestering virions.

The interaction of activated lymphocytes with infant tonsil epithelial cells sequestering HIV-1 initiates the spread of virus from epithelial cells to lymphocytes [3]. Binding of lymphocyte function-associated antigen-1 of activated lymphocytes to the intercellular adhesion molecule-1 of tonsil epithelial cells induces the disruption of cortical actin and the release of sequestered HIV-1 [3]. Thus, oral intraepithelial HIV-1 sequestration may contribute to the molecular pathogenesis of viral transmission from mucosal epithelial cells to virus-susceptible immune cells.

Antiviral innate immune proteins, including hBD-2 and -3, may play a critical role in the inactivation of intravesicular HIV-1 in infant oropharyngeal epithelium, thereby preventing HIV MTCT at the initial stage. Cell surface heparan sulfate proteoglycans in tonsil epithelium facilitate the simultaneous binding of HIV gp120 and hBD-2 and -3 and cointernalize HIV and hBD-2 and -3 into endosomes, where hBDs inactivate the virus [4]. HIV sequestration occurs in hBD-2 and -3–negative infant tonsil epithelial cells but not in hBD-2 and -3–positive adult epithelial cells [1]. Thus, hBD/HIV cointernalization in adult tonsil epithelial cells may disrupt the proteins of intravesicular virions and eliminate sequestered virions. The lack of hBD-2 and -3 expression in fetal/infant oral epithelial cells may permit viral sequestration in the endosomes.

To inactivate intravesicular virions, hBDs should be delivered into vesicles that contain HIV, including MVB and vacuoles. To achieve the higher level of penetration of hBD-2 and -3 into vesicles containing HIV-1, we used synthetic hBDs containing the unique motif YGRKKRRQRRR, which may promote hBD internalization into cells and vesicles. This motif has been identified in the HIV-1 regulatory protein Tat and is known as a protein transduction domain (PTD) [19,20,21,22]. The PTD signal is located between 47 and 57 amino acid residues of HIV-1 Tat’s basic domain. It contains arginine and lysine residues, which are positively charged and play a critical role in the penetration of Tat into cells [19]. A similar peptide has also been found in the VP22 protein of herpes simplex virus-1 [23]. The PTD peptides rapidly penetrate mammalian cells and tissues and are therefore called cell-penetrating peptides (CPP). The PTD signal has been used to promote the delivery of various proteins into cells and tissues [24,25,26,27]. However, the molecular mechanism of PTD-mediated penetration of proteins into mammalian cells is not fully understood. It has been shown that the PTD may initiate endocytosis and macropinocytosis [28,29,30,31,32,33,34,35], which facilitate internalization of cargo proteins tagged with PTD. Furthermore, PTD could induce the penetration of proteins directly via cell membranes [34,35,36,37,38]. Direct penetration is energy independent and may occur at a low temperature [39,40,41,42]. It is also possible that PTD-mediated internalization of cargo proteins is facilitated by a combination of endocytosis, macropinocytosis, and direct penetration [33,34,35].

In this report, we show that HIV-1 Tat’s PTD signal (i) facilitates the penetration of hBD-2 and -3 proteins into endosomes of infant tonsil epithelial cells containing HIV-1 and (ii) inactivates intravesicular HIV-1, which may reduce viral MTCT.

## 2. Materials and Methods

### 2.1. Ethics Statement

This study was conducted according to the principles expressed in the Declaration of Helsinki and was approved by the Committee on Human Research of the University of California, San Francisco (IRB approval # H8597-30664-03). All subjects provided written informed consent for the collection of tissue samples.

### 2.2. Viruses, Cells, and Tissues

Laboratory-adapted dual (X4-R5)-tropic HIV-1_SF33_ and the primary isolates R5-tropic HIV-1_SF170_ and X4-tropic HIV-1_92UG029_ were grown in peripheral blood mononuclear cells (PBMC). Before infection, PBMC were activated with 2.5 µg/mL phytohemagglutinin (Sigma, Burlington, MA, USA) and 1 µg/mL interleukin-2 (BD Biosciences, Franklin Lakes, NJ, USA) for 3 days. All stock viruses of HIV-1 were purified by using Amicon Ultra-15 columns as described [43,44]. Primary tonsil epithelial keratinocytes were expanded from tonsil tissue samples collected after routine tonsillectomies in HIV-negative children under 5 years of age. Mucosal epithelial layers were separated from subepithelial connective tissues, and epithelial cells were dissociated with 1 mg/mL collagenase (Life Technologies, Carlsbad, CA, USA) and 500 mg/mL dispase (STEMCELL Technologies, Vancouver, BC, Canada) for 5–10 h at 37 °C. The cell suspension was washed with KGM medium twice by centrifugation at 1200 rpm for 10 min. The pellet was dissociated into single cells in 0.05% trypsin/EDTA for 10–30 min. To remove debris, cells were filtered through a 100 µm membrane (BD Biosciences). Trypsin then was inactivated with KGM medium containing 10% fetal bovine serum, cells were washed, and keratinocytes were grown in KGM medium. The purity of the epithelial cells was verified by immunostaining of cells in a cocktail of antikeratin antibodies containing Ab-1 and Ab-2 (Thermo Fisher Scientific, South San Francisco, CA, USA); 100% of cells expressed keratin and were used in all experiments.

Polarized cells were established in 0.45 µm or 3 µm Transwell two-chamber filter inserts (Fisher Scientific, Waltham, MA, USA), as described in our previous work [1,45,46,47,48]. The polarity of epithelial cells was verified by immunodetection of the tight junction protein occludin and measurement of transepithelial resistance (TER) using a Millicell-ERS voltohmmeter, as described in our previous work [1,45,46,47]. To disrupt epithelial junctions, polarized cells were incubated with 10 mM EDTA for 1 h [2]. Cells were then used for TER measurement.

Tonsil tissues were collected from HIV-1-negative children under 5 years of age ~2 h after routine tonsillectomy. To establish polarized-oriented organ cultures, we placed tonsil explants (~5 × 5 mm) with mucosal epithelium, with the mucosal side facing up, in the upper chamber of Millicell filter inserts of 12 mm diameter and 0.4 µm pore size (Millipore, Burlington, MA, USA). The lateral edges of the explants were sealed with 3% agarose, as described [2,48]. The orientation of the explants was monitored by using a stereomicroscope (Stereomaster, Fisher Scientific, Waltham, MA, USA).

### 2.3. Generation of Synthetic hBD-2 and hBD-3

Synthetic hBD-2 and -3 were generated at Pierce Biotechnology, Inc., (Waltham, MA, USA) by custom order. The following peptides were generated by chemical synthesis using solid-phase peptide synthesis:

1. hBD-2: MRVLYLLFSFLFIFLMPLPGVFGGIGDPVTCLKSGAIC;
2. HPVFCPRRYKQIGTCGLPGTKCCKKP;
3. hBD-2 tagged with HIV-1 Tat PTD sequence: YGRKKRRQRRR -MRVLYLLFSFLFIFLMPLPGVFGGIGDPVTCLKSGAICHPVFCPRRYKQIGTCGLPGTKCCKKP;
4. hBD-3: GIINTLQKYYCRVRGGRCAVLSCLPKEEQIGKCSTRGRKCCRRKK;
5. hBD-3 tagged with HIV-1 Tat PTD sequence: YGRKKRRQRRR- GIINTLQKYYCRVRGGRCAVLSCLPKEEQIGKCSTRGRKCCRRKK.

The beta connectivity of disulfide bonds in highly purified proteins was independently verified by mass mapping of peptide fragments generated by trypsin digestion and Edman degradation.

### 2.4. Confocal Immunofluorescence Assay

Cells were fixed with 4% paraformaldehyde and 2% sucrose in PBS for 5 min, and then permeabilized with 0.01% Triton X-100 in 4% paraformaldehyde for 5 min. For immunostaining of EEA1 and rabankyrin, rabbit antibodies were used (both from Abcam) (1 µg/mL). For detection of LAMP1 and LBPA, 1 µg/mL mouse monoclonal antibodies (Santa Cruz Biotechnology and Millipore, respectively) were used. HIV-1 p24 was detected by using mouse and rabbit anti-p24 antibodies (NIH AIDS Research and Reference Reagent Program and Abcam) (5 µg/mL of each). For detection of immune cells, we used rabbit monoclonal antibodies to CD4, CD68, and CD1c, which are markers for CD4+ lymphocytes, macrophages, and dendritic cells (DC), respectively (1 µg/mL of each; all from Abcam). Secondary antibodies labeled with DyLight 488, DyLight 594, Alexa Fluor, and cyanine 5 (Cy5) were obtained from Jackson ImmunoResearch. Cell nuclei were stained with TO-PRO-3 iodide or DAPI (blue) (Molecular Probes, Eugene, OR, USA). The specificity of each antibody was verified by negative staining with the corresponding isotype control antibody. Cells were evaluated by using a Leica SP5 laser confocal microscope (Leica Microsystems, Wetzlar, Germany) or Nikon Eclipse E400 fluorescence microscope (Nikon, New York, NY, USA).

For quantitative analysis of HIV p24-positive immune cells, tonsil tissue sections were coimmunostained for p24 with CD68, CD4, and CD1c, which are markers for macrophages, lymphocytes, and DC, respectively. Macrophages, lymphocytes and DC, expressing HIV-1 p24 were counted in 10 randomly selected microscopic fields (x200) per section. At least three sections for each tonsil tissue explant were quantitatively evaluated. Results are presented as the average number of p24-positive immune cells per mm^2^.

### 2.5. Western Blot Assay

Cells were lysed with 1.0% Triton X-100 buffer (150 mM NaCl, 10 mM Tris/HCl, pH 8.0, and a cocktail of protease inhibitors) and proteins were separated on a 4–20% gradient SDS-polyacrylamide gel. For detection of hBD-2 and hBD-3, mouse and goat antibodies from Santa Cruz Biotechnology and R&D Systems were used. ECL Western blotting detection reagents (Amersham, Little Chalfont, UK) were used for visualization of protein bands. An equal protein load was verified by detection of β-actin (R&D Systems, Inc, Minniapolis, MN, USA). For detection of HIV gp120 and p24 proteins, mouse monoclonal antibodies ID6 and #24-2, respectively (both from the NIH AIDS Research and Reference Reagent Program), were used.

### 2.6. hBD Penetration Assays

To examine penetration of hBDs, hBD-2 or -3—with or without PTD—was added to the apical surfaces of polarized cells, which were incubated for 1 h at 37 °C in the humidified incubator or 4 °C on ice. Cells were washed and trypsinized to remove uninternalized hBDs, and intracellular hBDs were detected by Western blotting. All experiments with hBDs were performed in serum-free KGM (Lonza, Basel, Switzeland). The toxicity of hBDs on polarized cells was examined after 24 h of incubation by using an MTT Cell Viability Assay Kit (Biotium, Inc., Fremont, CA, USA).

For hBD–HIV cointernalization, hBD-2 or -3 was added to the apical surface of polarized tonsil epithelial cells and incubated at 4 °C for 30 min as described in our previous work [4]. Cells were then washed, and HIV-1_SF33_ (20 ng/mL) was added for 2 h at 37 °C. Cells were then washed and trypsinized to remove uninternalized HIV, and intracellular HIV-1 was detected by ELISA p24.

For inhibition of acidification of intravesicular compartments, cells were treated with non-toxic concentrations of NH_4_Cl (30 mM) or bafilomycin A1 (Sigma) (0.1 µM) for 1 h [4,49], and then cells were exposed to HIV-1_SF33_ for 4 h in the presence of NH_4_Cl or bafilomycin. For the next 4 h, cells were treated with hBD-2^PTD^ or hBD-3^PTD^, after which cells were examined for intracellular HIV-1_SF33_ by ELISA p24.

### 2.7. Assessing HIV-1 Sequestration in Polarized Epithelium

HIV-1 at 20 ng/mL was added to the apical surface of polarized tonsil epithelial cells, and cells were incubated at 37 °C in CO_2_ for 4 h [3,9]. Then, uninternalized virions were removed using mild 0.05% trypsin for 2–3 min at room temperature [2,50]. Trypsin was inactivated by KGM containing 10% FBS, and the integrity of cell polarity was confirmed by measuring TER of polarized cells throughout the experiment. Cells were maintained for 24 or 48 h and then trypsinized with 0.25% trypsin. Intracellular virus was detected by ELISA p24 (PerkinElmer, Waltham, MA, USA).

To analyze the penetration of HIV-1 into vesicular compartments of tonsil tissues, we added 100 ng of p24 HIV-1 per explant to the upper chambers (mucosal surface), and filter inserts with explants were incubated at 37 °C or 4 °C. Cross-sections of tissues were cut to 5 µm thickness with a horizontal orientation.

### 2.8. HIV Infectivity Assay

To determine the infectivity of intravesicular HIV-1, cells containing virus were incubated with mild trypsin for 2–5 min, and trypsin was then inactivated with medium containing 10% fetal serum. Cells were washed with PBS (pH 7.2) and homogenized by using motor-driven grinders connected with disposable pellet pestles (Pellet Pestle Cordless Motor, Kimble Kontes, South San Francisco, CA, USA) as described in our previous work [4]. Homogenates were centrifuged at 2000 rpm for 10 min, and supernatants were used for detection of virus. Bradford protein assay was used to determine the protein concentrations of supernatants and activated PBMC were infected with equal amounts of homogenate. After 5–7 days, viral infection was measured by ELISA p24.

HIV-1 at 1 ng/p24/explant was added to the mucosal surface of polarized-oriented tonsil tissue explants. Tissues were washed after 4 h and maintained with culture medium. Duplicated tissue explants were used for each experimental condition.

To determine the anti-HIV role of hBDs, cells or tissues containing sequestered HIV-1 were washed and incubated with hBDs at 37 °C for 2 or 4 h, respectively. Cells or tissues were then washed and trypsinized to remove uninternalized virions from the cell surface, homogenized, and evaluated for HIV-1 infection.

### 2.9. Assessment of HIV-1 Spread from Polarized Tonsil Epithelial Cells to PBMC

To examine HIV-1 spread from polarized tonsil epithelial cells to lymphocytes, lymphocytes were cocultivated with epithelial cells sequestering HIV-1, as described in our previous work [3,9]. For lymphocyte cocultivation with the apical surface of tonsil epithelial cells, we added activated allogenic PBMC to the upper chambers of Transwell filter inserts, where the polarized tonsil epithelial cells containing sequestered HIV-1 were grown. Under this condition, lymphocytes have direct contact with the apical surface of epithelial cells. After 4 h, lymphocytes were collected by pipetting and centrifuged for 10 min at 1200 rpm. PBMC were grown for 5 days and analyzed for HIV-1 infection by ELISA p24.

### 2.10. Electron Microscopy

For the fixation of cells, we used 2% glutaraldehyde, 4% formaldehyde in 0.1 M sodium cacodylate buffer, pH 7.3. Cells were then treated with 1% osmium tetroxide and 2% uranyl acetate. Cells were dehydrated with ethanol and embedded in Eponate 812 or EmBed 812 (Ted Pella Inc., Redding, CA, USA). Uranyl acetate (2%) was used for staining of ultrathin sections, which were examined at 120 kV in a JEOL JEM 1400 transmission electron microscope.

### 2.11. Statistical Analysis

Differences in p24 values of PBMC and epithelial cells infected with HIV in the presence or absence of hBD-2 or -3 were compared by using Student’s *t*-test. *p* values < 0.05 were considered significant. Results are expressed as mean ± SD.

## 3. Results

### 3.1. The PTD Signal Facilitates Internalization of hBD-2 and -3 Protein into Polarized Tonsil Epithelial Cells via Endocytosis and Direct Penetration

We hypothesized that the HIV-1 Tat PTD signal may facilitate high-level internalization of hBD-2 and -3 into polarized infant tonsil epithelial cells, including into endosomes containing HIV-1. This internalization may increase the antiviral effect of hBDs, leading to inactivation of intravesicular virions. To test this hypothesis, we generated synthetic hBD-2 and -3 and added PTD signal to the N terminus of hBDs (Pierce Biotechnology, Inc., Waltham, MA, USA). Oropharyngeal mucosal epithelia in vivo have a polarized organization [2,45]. Therefore, to establish a highly suitable model in vitro, the polarized tonsil epithelial cells (keratinocytes) from children under 5 years of age were propagated in two-chamber Transwell inserts, as described in our previous work [3,9].

**Figure 1:**
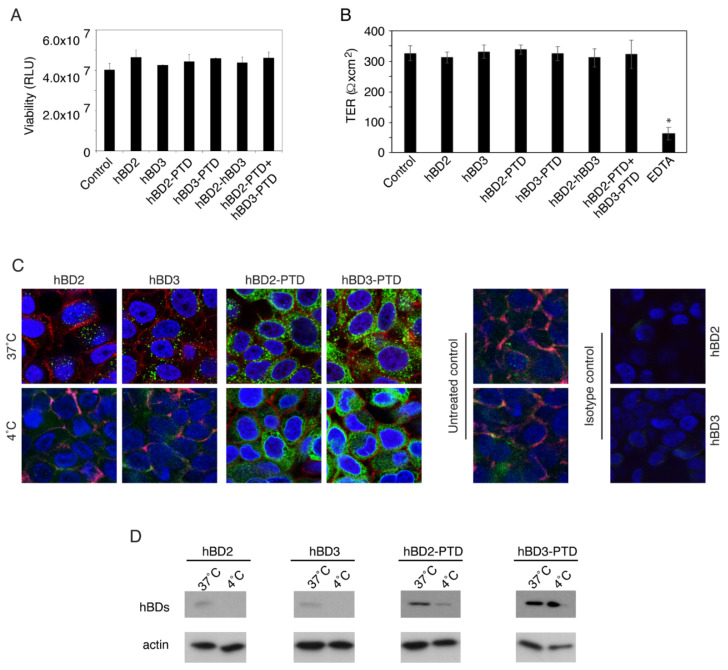
Protein transduction domain (PTD)-mediated penetration of polarized tonsil epithelial cells by hBD-2 and hBD-3. (**A**) hBD-2 and -3 proteins, with or without PTD signal, were added to the apical surface of polarized tonsil epithelial cells (100 µg/mL). Cells were also incubated with a combination of hBD-2 and -3 (50 µg/mL of each). After 24 h, cells were washed and examined for cell viability using a methyl tetrazolium (MTT) assay. RLU, relative light units. (**B**) Polarized tonsil epithelial cells treated with hBD-2 and -3, with or without PTD signal, were measured for transepithelial resistance (TER) using a Millicell-ERS voltohmmeter. One set of untreated cells was incubated with 10 mM EDTA for 1 h. (**C**) hBDs were added to the apical surface of polarized tonsil cells and incubated at 37 °C or 4 °C for 1 h. Untreated cells served as a control. Cells were immunostained for antibodies against hBD-2 and -3 (both in green) and occludin (red). Cells were analyzed by confocal microscopy. Nuclei were counterstained with DAPI (blue). Magnification: ×630. (**D**) Polarized cells treated with hBDs at 37 °C or 4 °C for 1 h were trypsinized and used for Western blotting with antibodies against hBD-2 and -3. (**A**,**B**) Results are shown as mean ± SD (*n* = 3). * *p* < 0.0001 compared with the untreated cells. (**A** through **D**) Similar results were obtained in two independent experiments using tonsil epithelial cells from independent donors.

To examine the potential toxic effect of hBDs on the polarized infant tonsil epithelial cells, we treated cells from their apical membranes independently with hBD-2 and -3 as well as with hBD-2^PTD^ and hBD-3^PTD^ (100 µg/mL each) for 24 h. This concentration of hBDs was chosen because of the physiological concentration of hBD-2 and -3 in oral epithelium, which may reach 100 µg in 100 µm-thick epithelial tissue [51]. Cells were also treated with a combination of hBD-2 and -3 or hBD-2^PTD^ and hBD-3^PTD^ (50 µg/mL each). Analysis of hBD-treated and untreated cells by the methyl tetrazolium (MTT) viability assay showed that none of the hBDs or their combination had a toxic effect on the polarized tonsil epithelial cells (Figure 1A).

hBD-treated polarized infant tonsil epithelial cells were also examined for transepithelial resistance (TER). TER measurement of hBD-treated and untreated polarized cells showed that none of the hBDs or their combination reduced the TER, in contrast to EDTA treatment, which drastically reduced TER (Figure 1B) by disruption of tight junctions [47]. Thus, these experiments showed that hBD-2 and -3 or their combination, with or without PTD signal, did not alter the polarity of infant tonsil epithelial cells.

To study the internalization of hBD-2 and -3 proteins, with or without PTD, through endocytosis and direct penetration, we added the hBDs to apical membranes of polarized tonsil epithelial cells incubated at 37 °C or 4 °C for 1 h. Cells were then fixed, and hBDs were detected by confocal immunofluorescence microscopy. In the cells incubated at 37 °C, the hBD-2 and -3 proteins without PTD signal were detected in cytoplasm, indicating their internalization. Intracellular localization of hBD-2 and -3 in these cells showed a punctate pattern of proteins in the perinuclei areas (Figure 1C), which is the typical pattern of endocytosis [52,53]. In contrast, hBDs without PTD signal in the cells incubated at 4 °C were not detected in the cytoplasm, indicating the absence of internalization (Figure 1C). This lack of hBD-2 and -3 internalization at 4 °C shows the inhibition of endocytosis, which is energy dependent and requires 37 °C [54,55].

Internalization of hBD-2^PTD^ and hBD-3^PTD^ was detected in the polarized tonsil cells incubated at 37 °C or 4 °C. Localization of internalized proteins in the cells incubated at 37 °C was in a punctate pattern, showing endocytosis of hBDs with PTD, which was higher than internalization of hBDs without PTD (Figure 1C). The hBD-2^PTD^ and hBD-3^PTD^ staining pattern in the cells incubated at 4 °C was mostly in a diffuse cytoplasmic pattern, indicating direct penetration of proteins due to PTD [42,52,53,56]. hBD-2 and hBD-3 immunostaining of untreated tonsil cells showed weakly positive or negative signals, consistent with our previous work [4].

Next, intracellular hBDs with or without PTD signal were detected by Western blot assay. Polarized tonsil cells were incubated with hBDs at 4 °C or 37 °C for 1 h. Cells were then dissociated with trypsin, which removes extracellular uninternalized hBDs [4]. Western blotting showed that hBD-2 and -3 without PTD were detected only in cells incubated at 37 °C; cells incubated at 4 °C did not contain intracellular hBD-2 and -3, indicating the lack of hBD internalization (Figure 1D). Western blotting of cells incubated with hBD-2^PTD^ and hBD-3^PTD^ showed that hBDs containing PTD signal were detected in both 37 °C and 4 °C incubations. Internalization of hBD-2^PTD^ and hBD-3^PTD^ at 4 °C indicates that proteins were penetrated by direct penetration, which does not require 37 °C. The amount of intracellular hBD-2^PTD^ and hBD-3^PTD^ proteins incubated at 37 °C was substantially higher than that of hBD-2 and -3 without PTDs. These data indicate that hBD-2^PTD^ and hBD-3^PTD^ internalization is due to both endocytosis and direct penetration.

### 3.2. The PTD Delivered hBD-2 and -3 to Pre-Existing Vesicles Containing HIV-1, Leading to Its Inactivation

**Figure 2:**
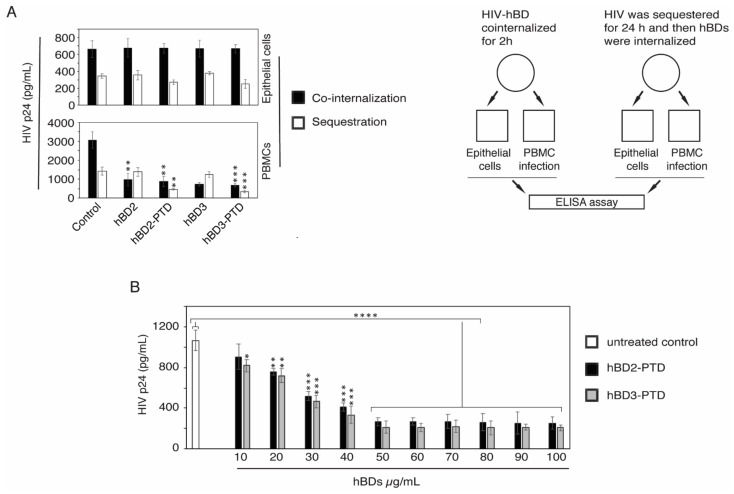
PTD does not change the anti-HIV function of hBD-2 and -3 proteins. (**A**, left panel) hBD-2 and -3, with or without PTD signal (100 µg/mL each), were added to the apical surface of polarized tonsil epithelial cells and incubated at 4 °C for 30 min. Cells were washed, HIV-1_SF33_ (20 ng/mL) was added to the apical surface, and cells were incubated at 37 °C for hBD–HIV cointernalization. After 2 h, cells were homogenized and samples were divided into two groups. In parallel experiments, tonsil cells were exposed to HIV-1_SF33_ (20 ng/mL) for viral sequestration; after 24 h, hBDs (100 µg/mL) were added to the apical surface of the cells for 2 h. Cells were then homogenized and divided into two groups. One group of samples was examined by ELISA p24, which shows intracellular HIV-1. Another group of samples was used for infection of PBMC; after 5 days, PBMC were examined by ELISA p24, which shows the infectivity of intraepithelial virus. The design of these experiments is shown in the schematic diagram (**A**, right panel). (**B**) Polarized tonsil epithelial cells were exposed to HIV-1_SF33_ (20 ng/mL); after 48 h, cells were treated with various concentrations of hBD-2^PTD^ and hBD-3^PTD^ for 2 h. Cells were then homogenized, and PBMC were infected with intraepithelial virions. After 5 days, viral infection in PBMC was examined by ELISA p24. (**A**,**B**) Results are shown as mean ± SD (*n* = 3) and were reproduced in two independent experiments. * *p* < 0.05, ** *p* < 0.01, *** *p* < 0.001, and **** *p* < 0.0001 compared with the untreated cells.

We have shown that cointernalization of hBD-2 and -3 with HIV-1 in polarized tonsil epithelial cells inactivates virions in the intraepithelial vesicles [4]. To examine if hBD-2 and -3 containing PTD can also inactivate vesicular HIV-1 by cointernalization of virions, we exposed polarized tonsil epithelial cells to HIV-1_SF33_ and to hBDs with or without PTD signal for 2 h. In parallel experiments, we exposed polarized tonsil epithelial cells to HIV-1_SF33_ for 48 h for viral sequestration and then exposed cells to hBDs with or without PTD signal for 2 h. Cells not exposed to hBD served as a control. Cells were then homogenized, and the infectivity of virus was evaluated in PBMC (Figure 2A). ELISA p24 showed that, in the cointernalization experiments, hBDs with or without PTD signal inactivated intraepithelial HIV-1_SF33_, indicating that coendocytosis of hBDs with or without PTD inactivates HIV-1 in the endosomes, i.e., PTD does not alter the antiviral activity of hBD-2 and -3. Analysis of the infectivity of virions sequestered in tonsil epithelial cells after 48 h showed a lack of viral inactivation by hBD-2 and -3 without PTD. In contrast, viral inactivation was clearly detected by hBD-2^PTD^ and hBD-3^PTD^, showing that PTD delivered the hBDs to pre-existing vesicles containing the virus.

To determine the optimal concentration of hBD-2^PTD^ and hBD-3^PTD^ for the highest viral inactivation, we exposed polarized tonsil epithelial cells containing sequestered HIV-1_SF33_ to increasing concentrations of hBDs (10–100 µg/mL) for 2 h (Figure 2B). Cells were then homogenized and examined for viral infectivity in PBMC. Data showed that the antiviral effect of hBDs started at 10 µg/mL, and the highest level was detected at 50–60 µg/mL, which reduced viral infectivity by ~80% (Figure 2B). HIV-1 infectivity was not reduced further at the highest concentration (60–100 µg/mL). These data also showed that internalization of hBD-2^PTD^ and hBD-3^PTD^ depends on their concentration, which indicates that PTD is mediated by direct penetration of proteins [57].

### 3.3. PTD-Mediated Internalization of hBD-2^PTD^ and hBD-3^PTD^ into MVB and Vacuoles, Which Sequester HIV-1

**Figure 3:**
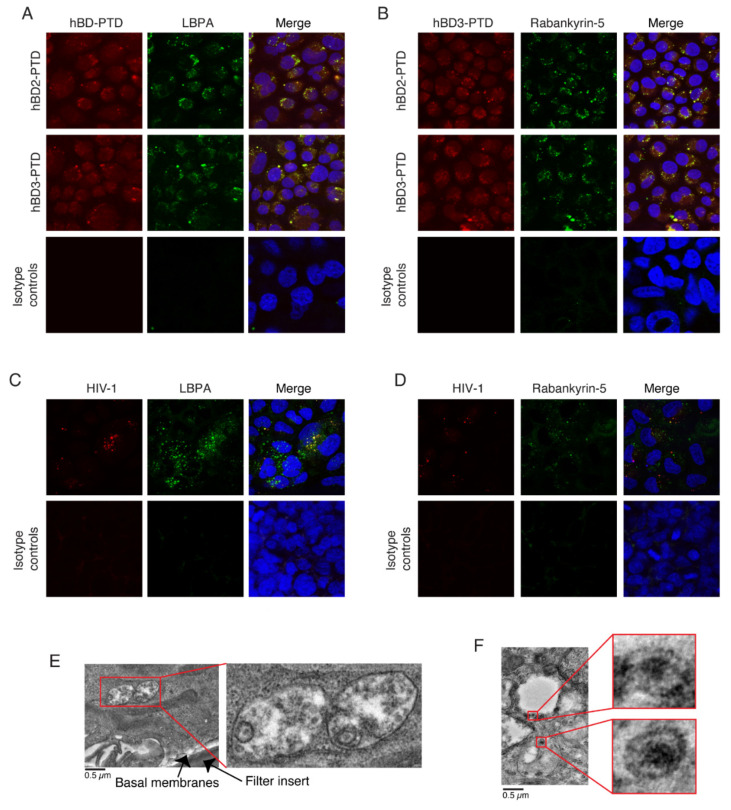
Colocalization of hBD-2^PTD^ and hBD-3^PTD^ with markers of MVB and vacuoles. Polarized tonsil epithelial cells were exposed to HIV-1_SF33_ (20 ng/mL); after 48 h, hBD-2^PTD^ and hBD-3^PTD^ (50 µg/mL each) were added to the apical surface of epithelial cells for 2 h. Cells were washed, fixed, and coimmunostained for hBD-2^PTD^ or hBD-3^PTD^ (both red) and for markers of MVB (**A**) and vacuoles (both green) (**B**). Cells were also costained for HIV-1 p24 (red) and for markers of MVB (**C**) and vacuoles (both green) (**D**). Cells were analyzed by confocal microscopy. Nuclei were counterstained with DAPI (blue). In merged panels, yellow shows colocalization of hBDs and HIV-1 with markers of MVB and vacuoles. (**A** through **D**) Magnification: ×630. One set of cells was fixed and analyzed by electron microscopy. Virions are shown in the MVB with multiple vesicles (**E**) or vacuoles (**F**). Magnification of insets: ×3–×8. Similar confocal and electron microscopic data were obtained in three independent experiments.

We have shown that, in tonsil epithelial cells, HIV-1 sequestration occurs mostly in the MVB and vacuoles [3,9]. To study the possible penetration of hBD-2^PTD^ and hBD-3^PTD^ into MVB and vacuoles, we treated polarized tonsil epithelial cells with hBD-2^PTD^ and hBD-3^PTD^ (50 µg/mL each) for 30 min. Cells were then coimmunostained with antibodies against hBD-2 and -3 and with lysobisphosphatidic acid (LBPA) and rabankyrin-5, which are markers for MVB and vacuoles, respectively. Confocal microscopy showed that both hBD-2^PTD^ and hBD-3^PTD^ were colocalized with LBPA and rabankyrin-5 (Figure 3A,B), indicating their penetration into MVB and vacuoles.

To detect sequestered HIV-1 in the MVB and vacuoles, we exposed polarized tonsil epithelial cells to HIV-1_SF33_ for 48 h and coimmunostained cells for HIV-1 p24 and LBPA or rabankyrin-5. Confocal microscopy showed that HIV-1 p24 was colocalized with markers of MVB and vacuoles (Figure 3C,D), indicating sequestration of virions in these vesicles. Transmission electron microscopy also showed the presence of mature virions in the MVB (Figure 3E) and vacuoles (Figure 3F). These findings are consistent with our previous work [3,9].

**Figure 4:**
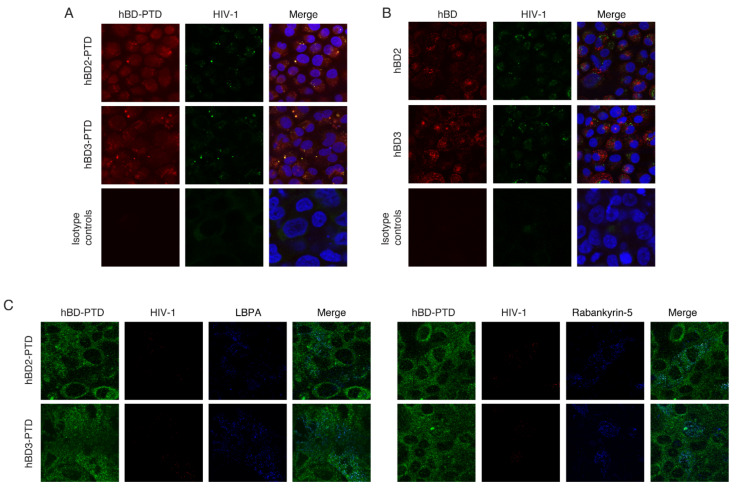
The PTD signal delivered hBD-2^PTD^ and hBD-3^PTD^ into vesicles containing HIV-1. (**A**,**B**) Polarized tonsil epithelial cells were exposed to HIV-1_SF33_ (20 ng/mL) and after 48 h, hBD-2 or -3 (50 µg/mL) was added with (**A**) or without (**B**) PTD for 1 h. Cells were then coimmunostained for hBD-2 or -3 (both red) and with HIV-1 p24 (green). Cells were analyzed by confocal microscopy. In merged panels, yellow shows colocalization of hBDs with HIV-1. (**C**) Cells were also coimmunostained for hBD-2 or -3 (green), p24 (red), and LBPA or rabankyrin-5 (blue), which are markers for MVB and vacuoles, respectively. White/pink in merged panels shows colocalization of hBDs, HIV-1, and markers of MVB or vacuoles. (**A**,**B**) Nuclei were counterstained with DAPI (blue). (**A** through **C**) Magnification: ×630. Similar data were obtained in three independent experiments.

In the next experiments, we examined PTD-mediated internalization of hBD-2^PTD^ and hBD-3^PTD^ into vesicles containing HIV-1. Polarized tonsil epithelial cells were exposed to HIV-1_SF33_ from the apical surface, and after 48 h, hBD-2^PTD^ and hBD-3^PTD^ as well as hBD-2 and -3 without PTD were added to the apical membranes; cells were incubated at 37 °C for 2 h. Cells were coimmunostained for HIV-1 p24 and hBD-2 or -3. Confocal microscopy of cells exposed to hBD-2^PTD^ and hBD-3^PTD^ showed that HIV-1 p24 colocalized with both hBDs, indicating that hBD-2^PTD^ and hBD-3^PTD^ were internalized into vesicles containing HIV-1_SF33_ (Figure 4A). In contrast, colocalization of HIV-1 p24 with hBD-2 and -3 was not detected in the cells exposed to hBD-2 and -3 without PTD (Figure 4B). These findings indicate that hBDs without PTD were internalized into cells, but they did not enter already-formed vesicles containing virions. Coimmunostaining of cells for HIV-1 p24 and hBD-2 or -3 with LBPA or rabankyrin-5 showed that virus in the MVB and vacuoles colocalized with hBDs, indicating the penetration of hBD-2^PTD^ and hBD-3^PTD^ into these vesicles containing virions (Figure 4C). Thus, PTD may play a critical role in the delivery of hBDs from cytosol into MVB and vacuoles by direct penetration via vesicular membranes.

### 3.4. PTD-Mediated hBD-2^PTD^ and hBD-3^PTD^ Internalization into Tonsil Epithelial Cells Inactivates Intravesicular HIV-1

To compare the antiviral role of hBD-2 and -3 with or without PTD signal, we exposed polarized tonsil epithelial cells from three independent donors to HIV-1_SF33_; after 48 h we added hBD-2 and -3, with or without PTD, independently or in combination. After 2 h, cells were homogenized, and intraepithelial virions were examined by ELISA p24. In parallel experiments, the infectivity of intraepithelial virions was examined in PBMC.

**Figure 5:**
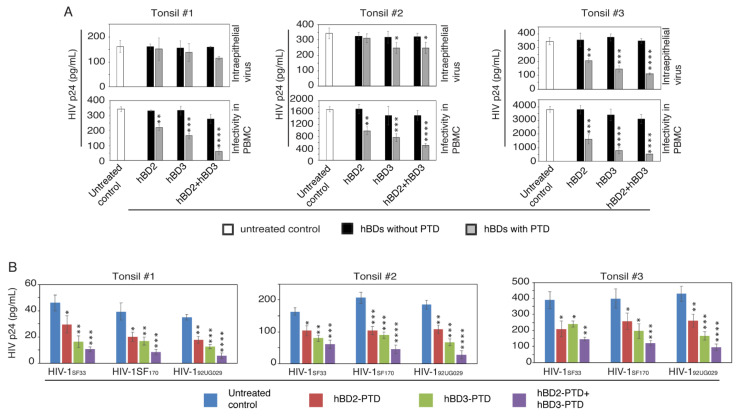
Internalization of hBD-2^PTD^ and hBD-3^PTD^ proteins into tonsil epithelial cells inactivated intravesicular HIV-1. (**A**) Polarized tonsil epithelial cells from three independent donors were exposed to HIV-1_SF33_ (20 ng/mL), and after 48 h, cells were treated with hBD-2 or -3 (50 µg/mL) with or without PTD signal for 2 h. One set of cells was treated with a combination of hBD-2 and -3 (50 µg/mL each). Cells were then homogenized, and samples were divided into two groups. One group was examined by ELISA p24, which showed intraepithelial HIV-1. The second group was used to infect PBMC; after 7 days, PBMC were examined by ELISA p24, which showed the infectivity of intraepithelial virus. (**B**) Polarized tonsil epithelial cells from three independent donors were exposed to HIV-1_SF33_, HIV-1_SF170_, or HIV-1_92UG029_ (20 ng/mL each); after 48 h, cells were treated with hBD-2 and -3 as described in panel A. Cell homogenates were used for infection of PBMC, and after 5 days, HIV-1 infection was examined by ELISA p24. Data are shown as mean ± SD (*n* = 3). * *p* < 0.05, ** *p* < 0.01, *** *p* < 0.001, **** *p* < 0.0001 compared with untreated cells.

These data show that tonsil epithelial cells from all three donors contained intracellular virus, indicating their sequestration (Figure 5A, upper panels). Exposure of cells to hBD-2 and -3 without PTD did not reduce p24 in the epithelial cells and PBMC, showing their lack of antiviral effect. Detection of p24 in tonsil epithelial cells exposed to hBD-2^PTD^ and hBD-3^PTD^ showed that intraepithelial p24 was significantly reduced in one of the tonsil cell cultures (Tonsil #3) by hBD-2^PTD^ (~40%), hBD-3^PTD^ (50%), and hBD-2^PTD^ + hBD-3^PTD^ (60%) (Figure 5, right upper panel). A lesser reduction in intraepithelial p24 was observed in tonsil cells from another donor by hBD-3^PTD^ and hBD-2^PTD^ + hBD-3^PTD^ (Tonsil #2) (Figure 5, middle upper panel).

Analysis of intraepithelial HIV-1_SF33_ infectivity in PBMC showed that infectious activity of virions was reduced in the tonsil epithelial cells of all three donors exposed to both hBD-2^PTD^ and hBD-3^PTD^ proteins (Figure 5A, lower panels); however, this reduction was not always dependent on the reduction in p24 in epithelial cells. This was particularly true for hBD-2^PTD^, which did not significantly reduce p24 in tonsil #2 and tonsil #3 epithelial cells but reduced viral infectivity in PBMC. Reduction in viral infectivity by hBD-2^PTD^ and hBD-3^PTD^ was 30–70% of that of cells not treated with hBDs. The highest level of viral inactivation was detected by the combination of hBD-2^PTD^ and hBD-3^PTD^, which reached ~90%. These findings demonstrate that PTD-mediated delivery of hBD-2 and -3 into endosomes containing virus substantially inactivates intravesicular virus.

Next, we compared the antiviral function of hBD-2^PTD^ and hBD-3^PTD^ in tonsil epithelial cells sequestering HIV-1 strains with different viral tropisms. Polarized tonsil epithelial cells from three independent donors were incubated with dual-tropic HIV-1_SF33_, R5-tropic HIV-1_SF170_, and X4-tropic HIV-1_92UG029_ strains for 2 days. Cells were then exposed to hBD-2^PTD^ and hBD-3^PTD^ proteins independently or in combination. After 2 h, cells were dissociated, homogenized, and examined for viral infectivity in PBMC. ELISA showed that hBD-2^PTD^- and/or hBD-3^PTD^-mediated inactivation in all three viral strains (Figure 5B). This finding was consistent in cells from all three donors. The highest level of HIV inactivation, regardless of its tropism, was detected in a combination of hBD-2^PTD^ and hBD-3^PTD^ proteins.

### 3.5. Inhibition of Lysosome Acidification Reduces HIV-1 Inactivation by hBD-2^PTD^ and hBD-3^PTD^

**Figure 6:**
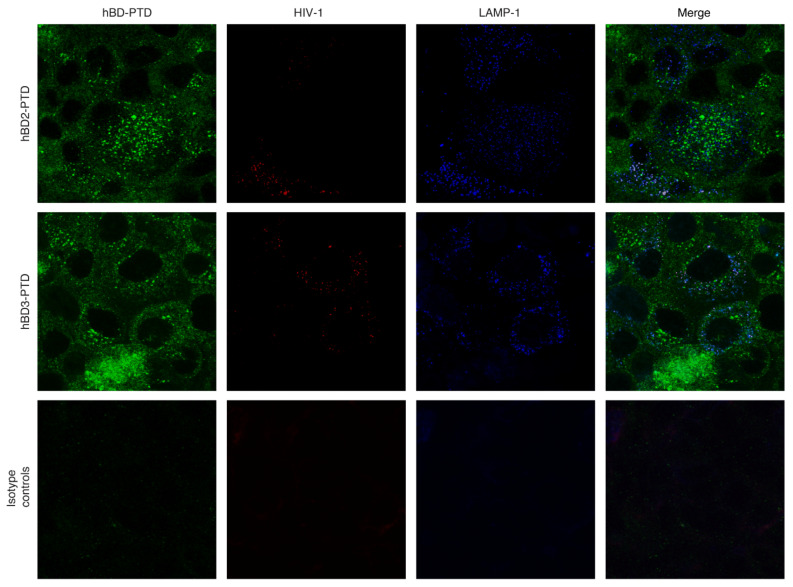
Colocalization of hBD-2^PTD^ and hBD-3^PTD^ proteins with HIV-1 in the lysosomes. Polarized tonsil epithelial cells were exposed to HIV-1_SF33_ (20 ng/mL); after 48 h, cells were treated with hBD-2 or -3 (50 µg/mL) with or without PTD signal for 2 h. Cells were fixed and immunostained for antibodies against hBD-2 or -3 (green), HIV-1 p24 (red), and lysosomal marker LAMP1 (blue). Cells were analyzed by confocal microscopy. Magnification: ×630. The white and pinkish-white color in the merged panels indicates colocalization of hBDs, p24, and LAMP1, indicating colocalization of hBDs and virions in the LAMP1-positive lysosomes. Results were reproduced in two independent experiments.

It has been shown that HIV-1 Tat PTD and other PTD signals may deliver the cargo proteins into lysosomes [35,58,59]. To study the possible mechanism of hBD-2^PTD^ and hBD-3^PTD^–induced HIV-1 inactivation, we exposed polarized tonsil epithelial cells to HIV-1_SF33_; after 24 h, cells were incubated with hBD-2^PTD^ and hBD-3^PTD^ proteins for 2 h. Cells were then coimmunostained for hBD-2^PTD^ or hBD-3^PTD^ and HIV p24 and lysosome-associated membrane protein (LAMP1). Confocal microscopy showed that both hBD-2^PTD^ and hBD-3^PTD^ were colocalized with p24 and LAMP1 (Figure 6). These data show that hBD-2^PTD^, hBD-3^PTD^, and HIV-1 were all delivered into lysosomes, showing that PTD may play a role in the fusion of MVB and vacuoles containing HIV-1_SF33_ with lysosomes.

**Figure 7:**
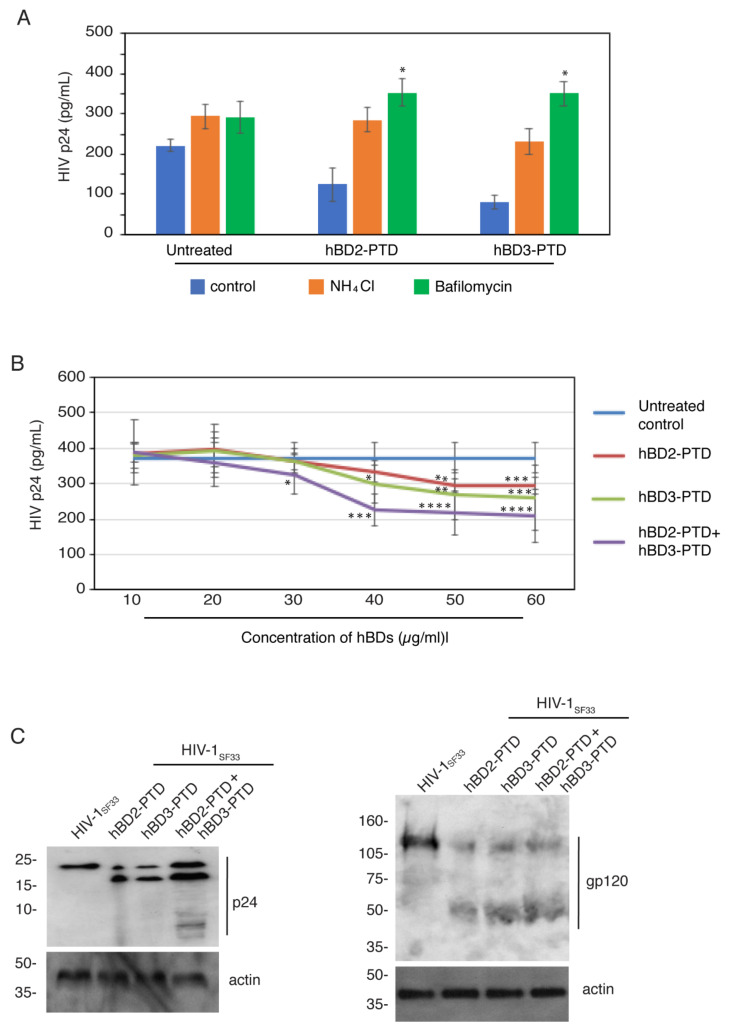
PTD signal may facilitate fusion of vesicles containing HIV-1 with lysosomes, which inactivate virions. (**A**) Polarized tonsil epithelial cells were pretreated with 30 mM ammonium chloride (NH_4_Cl) or 1 µM bafilomycin. After 1 h, cells were exposed to HIV-1_SF33_ for 4 h in the presence of NH_4_Cl and bafilomycin. During the next 2 h, cells were treated with hBD-2^PTD^ or hBD-3^PTD^ (50 µg/mL each) and cells were examined for intracellular HIV-1_SF33_ by ELISA p24. (**B**) Polarized tonsil epithelial cells were exposed to HIV-1_SF33_ (20 ng/mL); after 48 h, cells were treated with various concentrations of hBD-2^PTD^ or hBD-3^PTD^ or their combination (50 µg/mL each) for 2 h. Cells were then homogenized, and intracellular HIV-1 was examined by ELISA p24. (**C**) Polarized tonsil epithelial cells were exposed to HIV-1_SF33_ (100 ng/mL); after 24 h, cells were treated with hBD-2^PTD^ or hBD-3^PTD^ (50 µg/mL) or their combination (50 µg/mL of each) for 2 h. Cells were trypsinized and lysed, and intracellular virions were examined by Western blotting using antibodies against p24 and gp120. Results were reproduced in two independent experiments. (**A**,**B**) Results are shown as mean ± SD (*n* = 3). * *p* < 0.05, ** *p* < 0.01, *** *p* < 0.001, and **** *p* < 0.0001 compared with untreated cells.

In the next experiments, we pretreated polarized tonsil epithelial cells with 30 mM ammonium chloride (NH_4_Cl) or 0.1 µM bafilomycin, which are inhibitors of lysosomal acidification [4,60,61,62]. After 1 h, cells were exposed to HIV-1_SF33_ for 4 h in the presence of NH_4_Cl or bafilomycin. For the next 4 h, cells were treated with hBD-2^PTD^ or hBD-3^PTD^, after which cells were examined for intracellular HIV-1_SF33_ by ELISA p24. The results showed that the antiviral effect of hBD-2^PTD^ or hBD-3^PTD^ was eliminated in both NH_4_Cl- and bafilomycin-treated cells (Figure 7A). This reveals that the acidification of lysosomes plays a functional role in viral inactivation. The antiacidic effect of bafilomycin and the recovery of viral infectivity were stronger in hBD-2^PTD^- or hBD-3^PTD^-treated cells than in untreated cells. Thus, PTD may play a functional role in the fusion of vesicles containing HIV-1 and hBDs with the lysosomal compartments where virus was inactivated.

### 3.6. hBD-2^PTD^ and hBD-3^PTD^ Induce the Destabilization of HIV-1 p24 and gp120 Proteins of Intraepithelial Virions

Figure 5A (upper panels) shows that hBD-2^PTD^, hBD-3^PTD^ and hBD-2^PTD^ + hBD-3^PTD^ reduce HIV-1 p24 of intraepithelial virions, suggesting that hBDs may cause the degradation of p24. To examine the role of hBD-2^PTD^ and hBD-3^PTD^ in the degradation of HIV-1 p24, we exposed polarized epithelial cells containing endosomal HIV-1_SF33_ to various concentrations of hBD-2^PTD^ and hBD-3^PTD^ independently or in combination for 2 h. Analysis of intraepithelial virions by ELISA p24 showed that 40–60 µg/mL of hBD-2^PTD^ and hBD-3^PTD^ reduced p24 by ~20 and 25%, respectively (Figure 7B). The combination of hBD-2^PTD^ and hBD-3^PTD^ reduced p24 by 50–55%, suggesting that hBD-2^PTD^ and hBD-3^PTD^ may cause the disruption of p24. To test this possibility, intraepithelial p24 was examined by Western blotting. Exposure of tonsil epithelial cells containing HIV-1_SF33_ to hBD-2^PTD^ and/or hBD-3^PTD^ resulted in cleavage of the p24-generated additional protein band to 18 kDa (Figure 7C, left panel). Cells treated with a combination of hBD-2^PTD^ and hBD-3^PTD^ also generated 10, 8, and 6 kDa of small fragments of p24. This result indicates that hBD-2^PTD^ and/or hBD-3^PTD^ cause degradation of p24, which may reduce the infectivity of virions.

Figure 5A (upper panels) also shows that the hBD-2^PTD^- and hBD-3^PTD^-mediated reduction in viral infectivity in PBMC was partially or fully independent of the reduction in p24 in epithelial cells by these hBDs, particularly hBD-2. This could be the result of hBD-dependent disruption of gp120, an envelope protein of HIV-1. To examine the integrity of gp120 proteins, tonsil cells containing intravesicular HIV-1_SF33_ were exposed to hBD-2^PTD^ and/or hBD-3^PTD^ and after 2 h, gp120 was detected by Western blotting. Results showed that gp120 had two bands: 120 kDa and 50 kDa. The 50-kDa band was smeared (Figure 7C, right panel). In contrast, the untreated cells showed only one band at 120 kDa. These data clearly indicate that hBD-2^PTD^ and/or hBD-3^PTD^ disrupt the gp120 protein in the vesicles, generating one additional protein band.

Our findings indicate that hBD-2^PTD^ and hBD-3^PTD^ disrupt envelope and capsid proteins of intravesicular HIV-1 by their cleavage, which may occur in the lysosomal compartments because of PTD-facilitated fusion of MVB and vacuoles containing virus and hBDs with the lysosomes.

### 3.7. hBD-2^PTD^- and hBD-3^PTD^-Mediated Inactivation of Intraepithelial HIV-1 Reduces Viral Spread from Epithelial Cells to Lymphocytes

**Figure 8:**
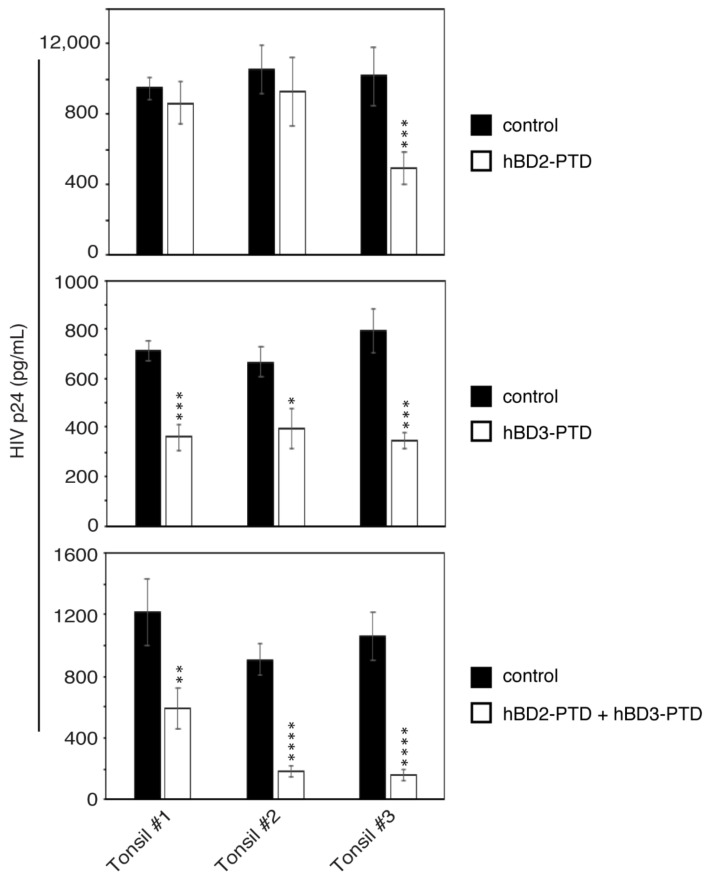
PTD-mediated hBD-2 and -3 internalization into tonsil epithelial cells containing HIV-1 reduced virus spread from epithelial cells to PBMC. Polarized tonsil epithelial cells from three independent donors were exposed to HIV-1_SF33_. After 48 h, epithelial cells containing intracellular virus were treated with hBD-2^PTD^ and hBD-3^PTD^ for 2 h; cells were washed and cocultivated with activated PBMC for the next 4 h. PBMC were then collected and cultured for 5 days, and viral infectivity was examined by ELISA p24. Results are shown as mean ± SD (*n* = 3). * *p* < 0.05, ** *p* < 0.01, *** *p* < 0.001, and **** *p* < 0.0001 compared with untreated cells.

In our previous work we showed that the direct interaction of PBMC and CD4+ T lymphocytes with tonsil epithelial cells containing intravesicular HIV-1 leads to the release of sequestered virions from epithelial cells and their spread to lymphocytes [3,9]. This mechanism may serve as a critical step for initiation of HIV MTCT. Therefore, we examined the role of hBD-2^PTD^ and hBD-3^PTD^ in HIV-1 spread from tonsil epithelial cells into lymphocytes. To test if hBD-2^PTD^ and hBD-3^PTD^ reduce the spread of HIV-1 from epithelial cells to lymphocytes, we exposed polarized tonsil epithelial cells from three independent donors to HIV-1_SF33_. After 48 h, epithelial cells containing intracellular virus were treated with hBD-2^PTD^ and/or hBD-3^PTD^ for 2 h and then tonsil cells were cocultivated with activated PBMC for the next 4 h. PBMC were then collected and cultured for 5 days, and viral infectivity was examined by ELISA p24. In hBD-2^PTD^-treated tonsil cells from one donor (tonsil #3), the spread of HIV-1_SF33_ from epithelial cells to lymphocytes was reduced by ~45% (Figure 8, top panel). In hBD-3^PTD^-treated tonsil cells from three donors, HIV-1_SF33_ spread was inhibited by 35–40%. Tonsil cells from all three donors exposed to a combination of hBD-2^PTD^ and hBD-3^PTD^ also showed inhibition of virus spread from epithelial cells to lymphocytes. Inhibition of viral spread from one donor’s tonsil cells was 50%, and in two others was ~90%. Notably, hBD-2^PTD^ treatment in cells from one donor (tissue #2) did not show significant inhibition of viral spread, whereas hBD-3^PTD^ treatment showed 35% inhibition. However, a combination of hBD-2^PTD^ and hBD-3^PTD^ in tissue #2 reduced viral spread by 90%. A similar trend was observed in tonsil cells from tissue #3. These data clearly demonstrate that hBD-2^PTD^- and/or hBD-3^PTD^-inactivated intraepithelial HIV-1_SF33_ leads to reduction in viral spread from epithelial cells to lymphocytes. The combination of hBD-2^PTD^ and hBD-3^PTD^ has a higher antiviral effect than does hBD-2^PTD^ or hBD-3^PTD^ alone and strongly inhibits viral spread from epithelial cells to lymphocytes. Thus, PTD-mediated penetration of hBD-2 and -3 into tonsil epithelial cells containing HIV-1 may play a critical role in reduction in HIV MTCT.

### 3.8. HIV Localization in Endosomes of Tonsil Mucosal Epithelial Tissues

**Figure 9:**
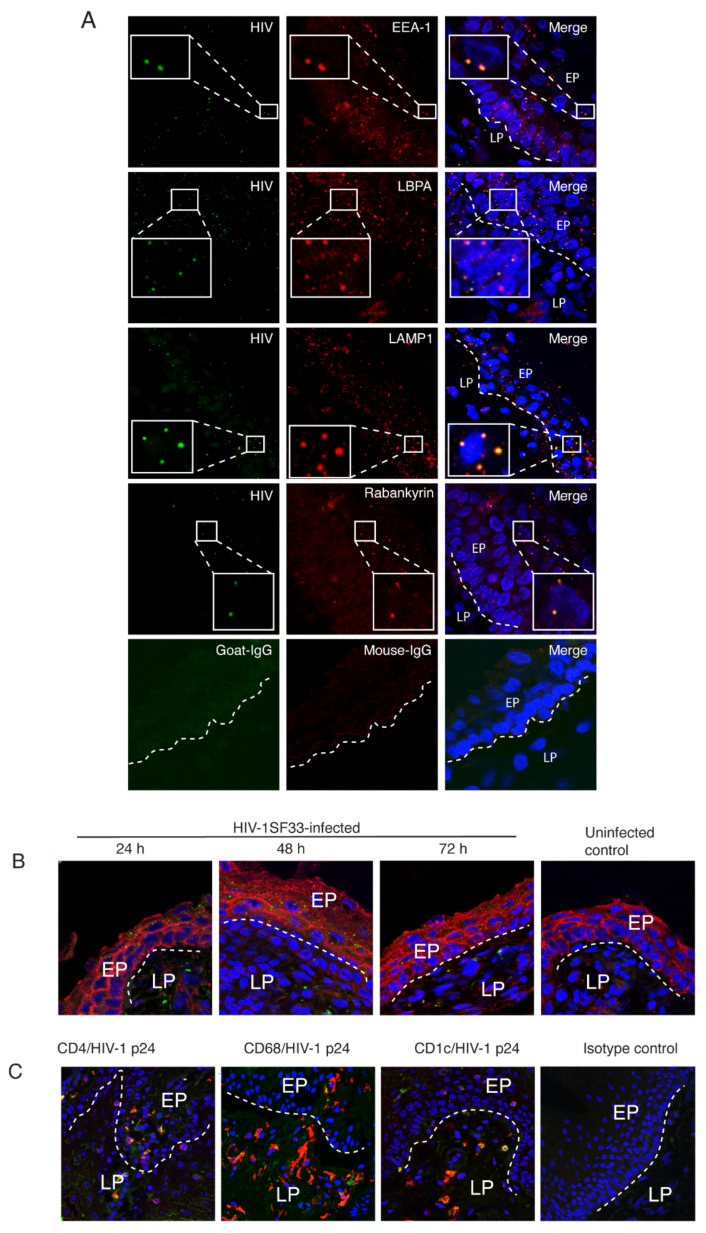
Penetration of HIV-1 into endosomal compartments of tonsil epithelium. (**A**) HIV-1_SF33_ (100 ng/mL) was added to the mucosal surface of polarized-oriented tonsil explants, and after 4 h, one set of tissues was fixed, sectioned, and costained for HIV-1 p24 (green) and endosome markers EEA1, LAMP1, LBPA, and rabankyrin (red). (**B**) Polarized-oriented tonsil explants were infected with HIV-1_SF33_ from the mucosal surface. Uninfected tissues served as a control. After 24, 48, and 72 h, tissue sections were coimmunostained for HIV-1 p24 (green) and occludin (red). (**C**) HIV-1_SF33_-infected tonsil tissue explants were maintained for 5 days, and sections were costained for HIV-1 p24 (green) and for markers of CD4 T lymphocytes, macrophages, and DC (red). Tissue sections were analyzed by confocal microscopy. Cell nuclei were counterstained in blue. EP, epithelium; LP, lamina propria. In merged panels, yellow indicates colocalization of HIV-1 p24 with endosome markers or markers of T lymphocytes, macrophages, and DC. Magnification: × 400. Results were reproduced in two independent experiments.

To examine HIV penetration into endosomes of tonsil epithelial tissues, we propagated polarized-oriented ex vivo tissue explants from palatine tonsil collected from three uninfected children under 5 years of age, as described in our previous work [2,45,48]. We then added HIV-1_SF33_ to the mucosal surface of polarized-oriented tonsil epithelial explants. After 4 h, we coimmunostained tissue sections for HIV and markers of early endosomes (EEA1), late endosomes (LAMP1 and LBPA), and vacuoles (rabankyrin-5). Confocal microscopy showed that HIV penetrated tonsil epithelium and colocalized with all four markers (Figure 9A). These data reveal HIV intravesicular localization of infant tonsil mucosal tissue, which is consistent with our findings in monostratified polarized epithelial cells (Figure 3, Figure 4, and Figure 6) [3,9].

Next, we added HIV-1_SF33_ to the mucosal surface of tonsil explants and examined one set of tissues for HIV-1 infection by p24 immunofluorescence assay after 24, 48, and 72 h. Confocal microscopy showed that a dot-like vesicular staining pattern of p24 was detected in the mucosal epithelial cells at 24 h after inoculation of virus; a similar vesicular pattern was detected at 48 and 72 h (Figure 9B). This indicates that intraepithelial HIV-1_SF33_ was present for a relatively long time, i.e., virions were sequestered in the vesicles of infant mucosal epithelium.

One set of HIV-1_SF33_-infected tissue explants was maintained for 5 days, after which tissue sections were examined for HIV-1-infected immune cells. Tissue sections were coimmunostained with antibodies against HIV-1 p24 and CD4, CD68, or CD1c, which are markers for CD4+ T lymphocytes, CD68+ macrophages, and CD1c+ DC, respectively. HIV-1 p24 was colocalized with CD4, CD68, and CD1c, indicating that CD4+ T lymphocytes, CD68+ macrophages, and CD1c+ DC in the tonsil tissues were infected with HIV-1_SF33_ (Figure 9C). These data indicate that HIV-1 had spread from tonsil epithelial cells to intraepithelial and subepithelial virus-susceptible cells.

### 3.9. hBD-2^PTD^ and hBD-3^PTD^ Reduced HIV-1 Spread in Ex Vivo Infant Tonsil Tissues

Beta-galactosidase fusion protein with HIV-1 Tat PTD mediates the rapid spread of beta-galactosidase in the liver, heart, and other tissues of mice [63]. The spread of beta-galactosidase is not observed when only enzyme is administered [26,27]. Furthermore, previously we have shown that PTD containing the full-size HIV-1 Tat protein penetrates ex vivo buccal stratified epithelium [45]. Therefore, we hypothesized that hBD-2^PTD^ or hBD-3^PTD^ may penetrate tonsil epithelium and inactivate intravesicular HIV-1.

**Figure 10:**
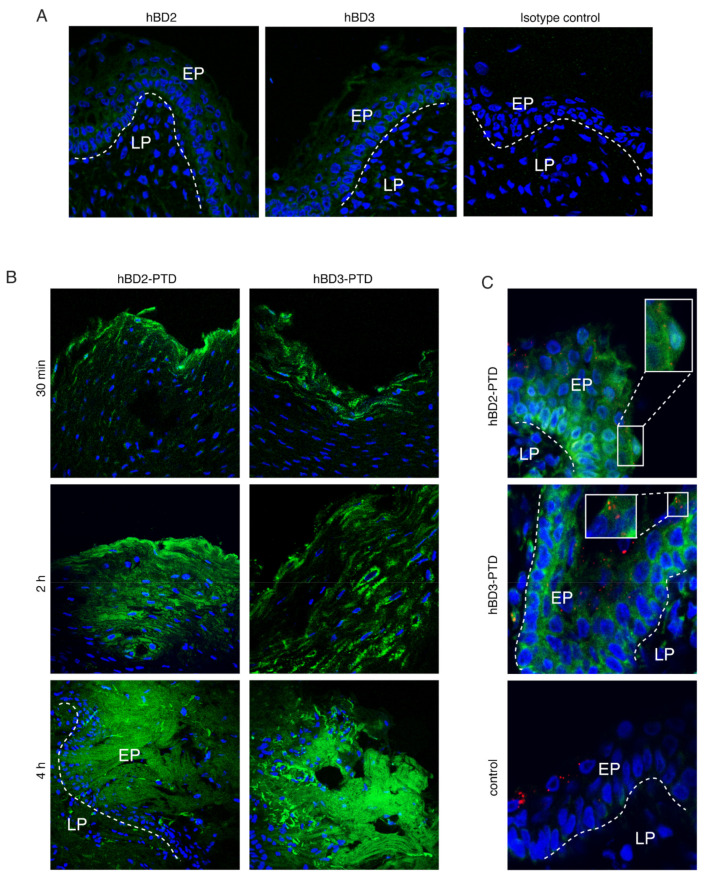
hBD-2^PTD^ and hBD-3^PTD^ internalization into ex vivo tonsil epithelial tissues. (**A**) Tonsil tissues were immunostained for hBD-2 and -3 (green). (**B**) Tonsil tissue explants were treated with hBD-2^PTD^ and hBD-3^PTD^ (100 µg/mL each), and after 30 min, 2 h, and 4 h, explants were fixed and immunostained for hBD-2 and -3. (**C**) Tonsil tissue explants were exposed to HIV-1_SF33_ (100 ng/mL) and after 4 h were treated with hBD-2^PTD^ and hBD-3^PTD^ for the next 4 h. Tissues were coimmunostained for HIV-1 p24 (red) and hBD-2 or -3 (green). In merged panels, yellow indicates colocalization of HIV-1 p24 with hBD-2^PTD^ and hBD-3^PTD^. (**A** through **C**) Cells were analyzed by confocal microscopy. Cell nuclei were counterstained in blue. EP, epithelium; LP, lamina propria. Magnification: × 400. Similar data were obtained in two independent experiments.

To examine the expression of endogenous hBD-2 and -3 in infant tonsil tissues, tonsil sections from three independent donors were immunostained for hBD-2 and -3. Confocal microscopy of tissue sections from all three donors showed that both hBDs have a low level of expression (Figure 10A), which is consistent with our earlier work [1].

To determine hBD-2^PTD^ or hBD-3^PTD^ penetration in tonsil tissues, we treated tissue explants from the mucosal surface with hBD-2^PTD^ or hBD-3^PTD^ (100 µg/mL each); after 30 min, 2 h, and 4 h, one set of tissue explants was fixed and examined for hBD penetration by immunofluorescence assay. Untreated tissues served as a control. Confocal microscopy showed that hBD-2^PTD^ or hBD-3^PTD^ penetrated the upper layers of stratified tonsil epithelium after 30 min of incubation and gradually increased penetration of the lower layers, covering almost the entire epithelium after 2 and 4 h of incubation (Figure 10B). However, hBDs were not distributed uniformly within the tissues. Penetration in some areas was more efficient than that in other areas. Penetration of hBD-2^PTD^ and hBD-3^PTD^ was detected in tonsil tissues from three independent donors.

To examine the penetration by hBD-2^PTD^ and hBD-3^PTD^ of HIV-1-infected tonsil tissues, explants were infected with HIV-1_SF33_ and after 4 h were treated with hBD-2^PTD^ or hBD-3^PTD^. After the next 4 h, tissues were fixed and coimmunostained for hBDs and HIV p24. Confocal microscopy showed that hBDs were colocalized with HIV-1 p24 in areas where hBD-2^PTD^ and hBD-3^PTD^ had penetrated (Figure 10C), indicating that hBDs were delivered into vesicles containing HIV-1.

**Figure 11:**
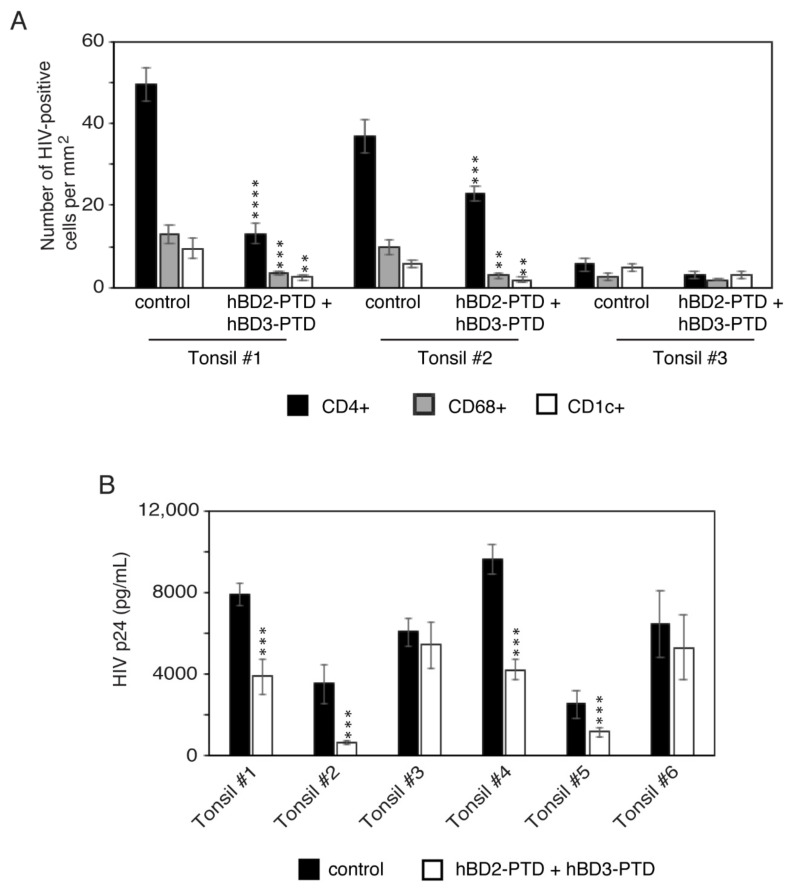
hBD-2^PTD^ and hBD-3^PTD^ internalization into tonsil epithelial tissues reduced HIV-1 spread from epithelial cells to CD4+ T lymphocytes, CD68+ macrophages, and CD1c+ DC. (**A**) Tonsil explants from three independent donors were infected with HIV-1_SF33_ p24 (1 µg/mL) and after 4 h, tissues were treated with hBD-2^PTD^ and hBD-3^PTD^ in combination (50 µg/mL each). After 2 days, tissue medium was changed for medium containing fresh hBDs; at day 5 after infection, tissues were fixed. Tissue sections were costained for HIV-1 p24 (green) and for markers of CD4 T lymphocytes, macrophages, and DC (red). HIV-1-infected CD4 T lymphocytes, macrophages, and DC were quantitatively evaluated and are presented as number of cells per mm^2^. (**B**) Tonsil explants from six independent donors were infected with HIV-1_SF33_ and treated with hBD-2^PTD^ and hBD-3^PTD^ as described in panel A. Five days later, culture medium was collected and tested for p24 using ELISA. p24 values were normalized per 50 mg of tissue. Explants treated with hBDs were compared with untreated explants. Results are shown as mean ± SD. ** *p* < 0.01, *** *p* < 0.001, and **** *p* < 0.0001.

To test the antiviral roles of hBD-2^PTD^ and hBD-3^PTD^ in HIV-1-infected tonsil tissues, explants from the mucosal surface were infected with HIV-1_SF33_; after 4 h, a combination of hBD-2^PTD^ and hBD-3^PTD^ was added to tonsil epithelium for 5 days. Untreated tissues served as a control. To detect HIV-1-infected target cells, we coimmunostained tissue sections for HIV p24 and CD4+ T lymphocytes, CD68+ macrophages, or CD1c+ DC. Quantitative analysis of HIV-1_SF33_-infected cells showed that hBD-2^PTD^ and hBD-3^PTD^ reduced the infection of CD4+ T lymphocytes, CD68+ macrophages, and CD1c+ DC by 20–70% compared with untreated tissues. hBD-2^PTD^- and hBD-3^PTD^-mediated inhibition of HIV-1_SF33_ was detected in the tonsil tissues from two of three donors (66.6%) (Figure 11A).

To evaluate the possibility of variation between donors, we infected tonsil explants from six independent donors with HIV-1_SF33_; after 4 h, tissue was exposed to a combination of hBD-2^PTD^ and hBD-3^PTD^. Untreated explants served as a control. After 5 days, culture medium was collected and tested by ELISA p24. Inhibition of HIV-1_SF33_ infection was detected in four of six tonsil tissues (60%) (Figure 11B). hBD-2^PTD^ + hBD-3^PTD^ mediated viral inhibition in three tonsil tissues (tonsils #1, #4, and #5) by ~50% and in one tonsil tissue (tonsil #2) by 80%. These data reveal that hBD-2^PTD^ and hBD-3^PTD^ may have a significant antiviral function in ex vivo infant tonsil tissues by reducing viral spread from epithelial cells into CD4+ T lymphocytes, CD68+ macrophages, and CD1c+ DC.

## 4. Discussion

We have shown that HIV-1 Tat protein’s PTD facilitates the penetration by hBD-2 and -3 innate proteins into infant tonsil epithelial cells, which contain sequestered virions in the MVB and vacuoles. Subsequently, PTD-tagged hBDs enter vesicles containing virions, leading to their inactivation.

In our previous work we showed that the cointernalization of hBD-2 and -3 with HIV-1 in the endosomes of tonsil epithelial cells induces the inactivation of intravesicular virions [4]. However, the lack of antiviral activity of hBD-2 and -3 in cells already containing sequestered HIV-1 in the endosomes indicates that newly internalized hBDs may not reach the pre-existing vesicles containing virions. In contrast, PTD-tagged hBD-2^PTD^ and hBD-3^PTD^ can reach the vesicles containing pre-existing virions and inactivate them.

Detection of hBD-2^PTD^ and hBD-3^PTD^ in the cytoplasm of tonsil epithelial cells with punctate and diffuse immunostaining patterns at 37 °C and 4 °C, respectively, suggested that PTD-mediated penetration by hBDs may have mechanisms of both endocytosis/macropinocytosis and direct penetration. These mechanisms would contribute to the highly efficient penetration by hBDs containing PTD signal compared with that by hBDs without PTD.

It is well known that HIV-1 Tat PTD-derived CPP can rapidly deliver various cargo, including proteins, DNA, RNA, and drug molecules, to cells via the plasma membrane in vitro and in vivo [64,65,66,67,68]. The molecular mechanism of these peptides’ penetration of cells varies significantly according to their properties, including physiochemical features, dose, incubation time, and cell type [33,34,35,69]. The internalization of Tat PTD-based CPP via cell membranes may occur by various mechanisms, such as clathrin- and caveolin-dependent or independent endocytosis, macropinocytosis, or direct penetration [28,31,32,52,53,56,70,71,72,73,74,75,76,77].

The mechanism of HIV-1 Tat’s PTD-mediated endocytosis, macropinocytosis and/or direct penetration of tonsil epithelial cells by hBD-2^PTD^ and hBD-3^PTD^ is not yet clear. Regardless of the molecular pathways of penetration by hBD-2^PTD^ and hBD-3^PTD^, both proteins have an antiviral effect on intravesicular virions.

**Figure 12:**
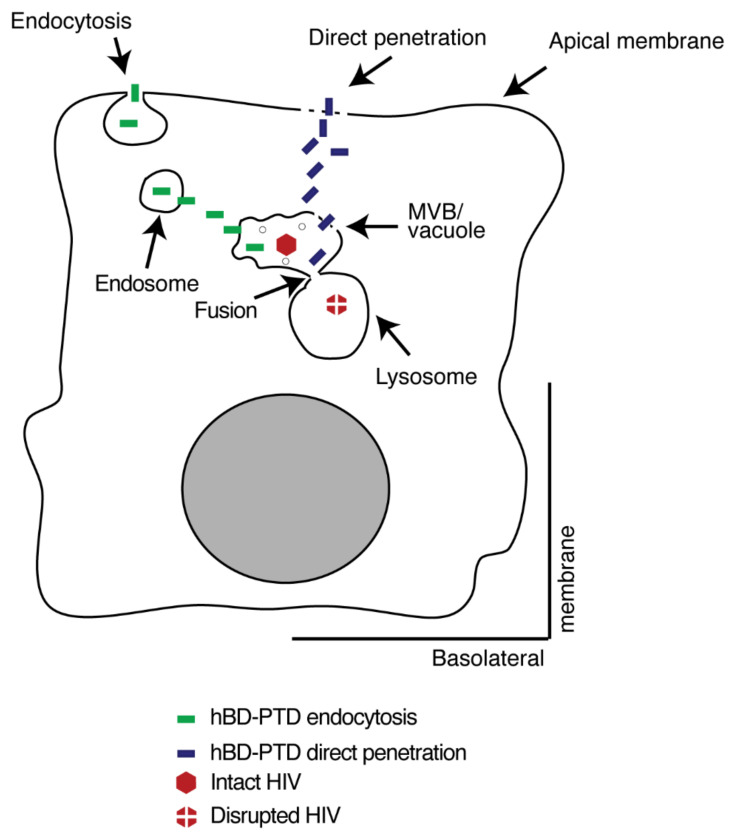
Model of hBD-2^PTD^- and hBD-3^PTD^-mediated inactivation of HIV-1 in tonsil epithelial cells. HIV-1 is internalized into polarized tonsil epithelial cells, and the majority of virions are sequestered in the MVB and vacuoles. hBD-2^PTD^ and hBD-3^PTD^ internalize into tonsil epithelial cells through endocytosis and direct penetration. hBD-2^PTD^ and hBD-3^PTD^ endocytosis generates endosomes containing hBDs, which are released from endosomes into cytosol and subsequently enter other endosomes, including those containing HIV-1, by direct penetration. hBD-2^PTD^ and hBD-3^PTD^ that directly penetrate via plasma membrane will also subsequently penetrate various vesicles, including MVB and vacuoles containing HIV-1. Vesicles containing HIV/hBD-2^PTD^/hBD-3^PTD^ may fuse with lysosomes by PTD, leading to the inactivation of intravesicular virions.

Detection of hBD-2^PTD^ and hBD-3^PTD^ in MVB and vacuoles containing HIV-1 indicates that PTD signal plays a critical role in delivering hBDs to the vesicular compartments containing virions. PTD-mediated delivery of hBDs into vesicles already containing virions may occur by the following mechanisms. First, penetration by hBD-2^PTD^ and hBD-3^PTD^ via endocytosis/macropinocytosis generates vesicles in cytoplasm that contain hBD, which subsequently may fuse with vesicles already containing virus (Figure 12). It has been shown that HIV-1 Tat PTD-derived CPP aggregate at phospholipid membranes, inducing the fusion of vesicles [29,78,79,80,81]. Thus, the newly generated vesicles with hBD-2^PTD^ and hBD-3^PTD^ may fuse with existing vesicles containing HIV-1 because of PDT-induced fusion pores [81]. Second, hBD-2^PTD^ and hBD-3^PTD^ may be released from newly formed vesicles into cytosol and subsequently enter existing vesicles, including those that contain HIV-1 (Figure 12). CPP may be released from endosomes into cytoplasm [82,83,84] and enter other vesicles [33,34,35,69]. PTD-mediated release of HIV-1 Tat from endosomes into cytosol has also been shown [85]. A third mechanism could be the direct penetration by hBD-2^PTD^ and hBD-3^PTD^ into cytoplasm and their subsequent entry into various endosomes, including vesicles containing HIV-1.

Colocalization of hBD-2^PTD^ or hBD-3^PTD^ and HIV-1 with a lysosome marker suggests that PTD may play a critical role in the fusion between endosomes containing HIV-1/hBD and lysosomes. HIV-1 Tat PTD-mediated delivery of galactocerebrosidase into lysosomes has been shown in 293 cells [58]. Inhibition of lysosomal acidification by ammonium chloride and bafilomycin decreases hBD-2^PTD^- and hBD-3^PTD^-induced inactivation of HIV-1. Bafilomycin inhibits autophagosome–lysosome fusion [60], and it is possible that bafilomycin also inhibits fusion of other vesicles with lysosomes, including MVB and vacuoles containing HIV-1.

Disruption of HIV-1 p24 and gp120 proteins by hBD-2^PTD^ and/or hBD-3^PTD^ indicates that hBDs may disintegrate these viral capsid and envelope proteins in the vesicles, which would explain the mechanism of hBD-2- and/or hBD-3-mediated inactivation of intravesicular virus shown in our previous work [1,4]. hBD-2-induced disruption of envelope of respiratory syncytial virus has also been shown [86], suggesting that human beta-defensins may have similar mechanisms against various viruses. Defensins bind to biological membranes and form multimeric pores, leading to the disruption of membranes [87,88,89,90,91,92,93].

Here, we showed that hBD-2^PTD^- and/or hBD-3^PTD^-mediated disruption of HIV-1 proteins may also occur by PTD-mediated fusion of vesicles containing HIV-1 with lysosomes, in which viral proteins may be digested by lysosomal enzymes. Thus, the PTD signal substantially increases the anti-HIV-1 functions of hBD-2 and/or hBD-3 in tonsil epithelial cells by delivering hBD-2 and -3 into vesicles containing virus and inducing their fusion with lysosomes.

Previously, we showed that HIV-1 Tat penetrates multistratified oral epithelium [45]. Our latest findings reveal that Tat’s PTD-facilitated penetration by hBD-2^PTD^ and/or hBD-3^PTD^ into polarized tonsil epithelial cells and tissue explants containing intravesicular HIV-1 induces viral inactivation. This in turn reduces viral spread from epithelial cells into virus-susceptible CD4+ T lymphocytes, CD68+ macrophages, and CD1c+ DC. The antiviral effect of hBD-2^PTD^ and/or hBD-3^PTD^ was independent of the tropism of HIV-1, suggesting that this approach may reduce HIV MTCT regardless of viral tropism [94,95,96,97].

It is also possible that hBD-2^PTD^ and/or hBD-3^PTD^ may inhibit HIV-1 infection in CD4+ T lymphocytes, CD68+ macrophages, and CD1c+ DC in the tonsil tissue. It has been shown that hBD-2 and/or hBD-3 may inhibit HIV-1 infection of CD4 T lymphocytes and macrophages [98,99,100,101].

The antiviral effect of hBD-3^PTD^ was higher than that of hBD-2^PTD^, which could be due to its C-terminal arginine-rich region (GKCSTRGRKCCRRKK) [102]. This region may serve as a CPP domain facilitating penetration of hBD-3 into mouse skin epithelium [102]. Thus, it is possible that the PTD-like domain of hBD-3 may promote its penetration into cells. In combination with Tat’s PTD, this may increase entry of hBD-3 into vesicles with HIV-1, inducing stronger viral inactivation and endosomal–lysosomal fusion than hBD-2^PTD^.

In summary, we have shown that HIV-1 Tat’s PTD plays a critical role in the delivery of antiviral innate proteins hBD-2 and -3 into vesicles of monostratified polarized infant tonsil epithelial cells and ex vivo tonsil tissue explants containing virus, leading to the inactivation of intraepithelial virions. hBD-2^PTD^ and/or hBD-3^PTD^-mediated HIV-1 inactivation reduces virus spread from epithelial cells to CD4+ T lymphocytes, CD68+ macrophages, and CD1c+ DC. Thus, this approach may help to design a new antiviral strategy to prevent or reduce HIV MTCT. HIV-1 sequestration also occurs in cervical and foreskin epithelial cells [3,9], and hBD-2^PTD^ and/or hBD-3^PTD^ may inactivate intravesicular HIV-1 in genital epithelial cells, which may reduce sexual transmission of HIV.

## Data Availability

Data are contained within the article.
